# Computing tensor Z-eigenpairs via an alternating direction method

**DOI:** 10.7717/peerj-cs.1242

**Published:** 2023-02-14

**Authors:** Genjiao Zhou, Shoushi Wang, Jinhong Huang

**Affiliations:** 1 Gannan Normal University, Ganzhou, China; 2 Gannan Normal University, Key Laboratory of Jiangxi Province for Numerical Simulation and Emulation Techniques, Ganzhou, China

**Keywords:** Higher-order tensor, Z-eigenvalues, Power method, Alternating direction method

## Abstract

Tensor eigenproblems have wide applications in blind source separation, magnetic resonance imaging, and molecular conformation. In this study, we explore an alternating direction method for computing the largest or smallest Z-eigenvalue and corresponding eigenvector of an even-order symmetric tensor. The method decomposes a tensor Z-eigenproblem into a series of matrix eigenproblems that can be readily solved using off-the-shelf matrix eigenvalue algorithms. Our numerical results show that, in most cases, the proposed method converges over two times faster and could determine extreme Z-eigenvalues with 20–50% higher probability than a classical power method-based approach.

## Introduction

The tensor eigenproblem has been of great interest since the seminal works of Qi (2005) and Lim (2005). It has numerous applications in several areas, including automatic control (Ni, Qi & Wang, 2008), magnetic resonance imaging (Qi, Yu & Wu, 2010; Qi, Yu & Xu, 2013; Schultz & Seidel, 2008), statistical data analysis (Zhang & Golub, 2001), image analysis (Zhang, Zhou & Peng, 2013), signal processing and navigation (Kofidis & Regalia, 2001; Ashourian & Sharifi-Tehrani, 2022; Sharifi-Tehrani & Sabahi, 2022; Sharifi-Tehrani, Sabahi & Danaee, 2021), and higher-order Markov chains (Li & Ng, 2014).

Unlike for matrices, there are several definitions of tensor eigenvalues and corresponding eigenvectors. For example, Qi (2005) proposed the definition of an H-eigenvalue and Z-eigenvalue as being equivalent to the *l*^*m*^-eigenvalue and *l*^2^-eigenvalue in Lim (2005), respectively. In Chang, Pearson & Zhang (2009), these definitions were unified by employing a positive definite tensor $\mathcal{B}$ while *m* was even. In this work, we mainly focus on computing Z-eigenvalues of symmetric tensors.

In general, the calculation of all eigenvalues of a higher-order tensor is very difficult due to the NP-hardness of deciding tensor eigenvalues over ℝ (Hillar & Lim, 2013). Fortunately, one only needs to compute the largest or smallest eigenvalue of a tensor in certain scenarios. For instance, to guarantee the positive definiteness of the diffusivity function in higher-order diffusion tensor imaging, we just need to compute the smallest Z-eigenvalue of the tensor and make sure it is nonnegative. In automatic control (Ni, Qi & Wang, 2008), the smallest Z-eigenvalue of a tensor is used to determine whether a nonlinear autonomous system is stable or not. According to the Perron–Frobenius theory, the spectral radius of a nonnegative tensor is the largest Z-eigenvalue of the tensor (Chang, Pearson & Zhang, 2008).

To obtain the extreme eigenvalues of a symmetric tensor, De Lathauwer, De Moor & Vandewalle (2000) introduced a symmetric higher-order power method (S-HOMP). However, it was pointed out by Kofidis & Regalia (2002) that the S-HOMP method is not guaranteed to converge while the objective function is not convex. To address this problem, Kolda & Mayo (2011) presented a shifted S-HOMP (SS-HOMP) for solving the eigenproblem, which is guaranteed to converge to a tensor eigenpair. A major limitation of SS-HOMP is the difficulty in selecting an appropriate shift. Hence, Kolda & Mayo (2014) further extended the SS-HOMP method to an adaptive version for computing extreme eigenvalues, called GEAP, which chooses the shift automatically.

Over the past few years, there has been extensive work on handling the extreme eigenvalue problem of symmetric tensors by solving different nonlinearly constrained models. Hu, Huang & Qi (2013) proposed a sequential semidefinite relaxations approach to compute extreme Z-eigenvalues. Han (2012) employed the BFGS method to solve an unconstrained optimization problem for finding real eigenvalues of even-order symmetric tensors. Cui, Dai & Nie (2014) computed all of the real Z-eigenvalues of symmetric tensors using a Jacobian semidefinite relaxation technique. Using the method proposed by Qi, Wang & Wang (2009) in which Z-eigenpairs are computed directly in a lower dimensional case, a sequential subspace projection method (SSPM) (Hao, Cui & Dai, 2015) was proposed to obtain the extreme Z-eigenvalues of symmetric tensors. All the methods mentioned above converge linearly or superlinearly. To speed up convergence, Jaffe, Weiss & Nadler (2018) presented a fast iterative Newton-based method that converges at a locally quadratic rate. Based on the idea of the SSPM method (Hao, Cui & Dai, 2015), Yu et al. (2016) proposed an adaptive gradient (AG) method in which an inexact line search, rather than an optimal stepsize, was adopted. The experimental results presented in Yu et al. (2016) showed that the AG method converges much faster and finds the extreme eigenvalues with a higher probability than those methods using power algorithms. For more related work, we refer readers to Benson & Gleich (2019), Chen, Han & Zhou (2016), Sheng & Ni (2021), Xiong et al. (2022), and references therein.

Despite the fact that the extreme eigenvalue problem has drawn a lot of attention in recent years, there are still some issues to address. For example, all algorithms mentioned above are not guaranteed to converge to the largest or smallest eigenvalue, which is exactly what we want to obtain in some applications (Chang, Pearson & Zhang, 2008; Ni, Qi & Wang, 2008), and instead only converge to an arbitrary eigenvalue of $\mathcal{A}$ depending on the initial conditions. However, in the case of symmetric matrices, those counterpart algorithms can always converge to the largest or smallest eigenvalue. Motivated by this, we propose to determine extreme eigenvalues by combining the method of solving matrix eigenvalue problems and tensor optimization techniques. To this end, we develop a novel method for computing the largest or smallest Z-eigenvalue of symmetric tensors using the variable splitting method.

The main contributions of this work are listed as follows:

• We reformulate a typical tensor Z-eigenvalue problem as an equivalent multi-variable linearly constrained problem using the variable splitting method, which has a structure similar to the matrix eigenvalue problem.
• We design an efficient algorithm to solve the reformulated problem, in which the tensor Z-eigenproblem is decomposed into a series of matrix eigenproblems that has been extensively studied and can be readily solved using off-the-shelf matrix eigenvalue algorithms.

The remainder of the article is organized as follows. In the next section, we formulate the tensor eigenproblem and introduce some classical methods for solving it. In Section 3, we propose a simple and efficient algorithm for the problem and analyze its convergence property. Section 4 reports some experimental results to show the efficiency of our proposed method. Finally, we conclude this article in Section 5.

## Problem Formulation and Related Work

### Problem formulation

An *m*th order *n*-dimensional real tensor consisting of *n*^*m*^ entries in ℝ:


\begin{eqnarray*}\mathcal{A}= \left( {a}_{{i}_{1}{i}_{2}\cdots {i}_{m}} \right) ,{a}_{{i}_{1}{i}_{2}\cdots {i}_{m}}\in \mathbb{R},1\leq {i}_{1},{i}_{2},\ldots ,{i}_{m}\leq n. \end{eqnarray*}

$\mathcal{A}$ is called symmetric if the value of *a*_*i*_1_*i*_2_⋯*i*_*m*__ is invariant under any permutation of its indices *i*_1_, *i*_2_, …, *i*_*m*_. For convenience, we use ${\mathbb{S}}^{ \left[ m,n \right] }$ to denote the set of all *m* th order *n*-dimensional real symmetric tensors.

Throughout this article, we use $\mathcal{A}{x}^{m&minus; k}(0&le; k&le; m)$ to simply denote a *k* th order *n*-dimensional tensor defined by

(1) \begin{eqnarray*}{ \left( \mathcal{A}{x}^{m-k} \right) }_{{i}_{1}{i}_{2}\cdots {i}_{k}}=\sum _{{i}_{k+1},\ldots ,{i}_{m}=1}^{n}{a}_{{i}_{1}{i}_{2}\cdots {i}_{k}{i}_{k+1}\cdots {i}_{m}}{x}_{{i}_{k+1}}\cdots {x}_{{i}_{m}},\end{eqnarray*}

*for* *all* 1 ≤ *i*_1_, *i*_2_, …, *i*_*k*_ ≤ *n* *and* 1 ≤ *k* ≤ *m*.

Obviously, $\mathcal{A}{x}^{m}$ is a scalar, $\mathcal{A}{x}^{m&minus; 1}$ is a vector, and $\mathcal{A}{x}^{m&minus; 2}$ is a matrix. For brevity, let $\mathcal{A}{x}^{p}{y}^{q}$ denote the result of $ \left( \mathcal{A}{x}^{p} \right) {y}^{q}= \left( \mathcal{A}{x}^{m-(m-p)} \right) {y}^{m-p-(m-p-q)}$, where 0 ≤ *p*, *q*, *p* + *q* ≤ *m*, and $\mathcal{A}{x}_{1}^{{p}_{1}}{x}_{2}^{{p}_{2}}\cdots {x}_{2}^{{p}_{k}}$ can be computed in a similar way, where 0 ≤ *p*_1_, *p*_2_, …, *p*_*k*_ ≤ *m*, and *k* is an arbitrary integer such that 0 ≤ *p*_1_ + *p*_2_ + ⋯ + *p*_*k*_ ≤ *m*.

Using the definition of Eq. (1), an *m*th degree homogeneous polynomial function *f*(*x*) with real coefficients can be represented by a symmetric tensor $\mathcal{A}$, *i.e.,* $f \left( x \right) =\mathcal{A}{x}^{m}$. We call $\mathcal{A}$ positive definite tensor if $\mathcal{A}{x}^{m}&gt; 0$ for all *x* ∈ ℝ^*n*^∖{0}. It is easy to understand that *m* must be even in this case.

As mentioned before, there are several definitions of tensor eigenvalues and corresponding eigenvectors. In this work, we mainly focus on computing Z-eigenvalues of symmetric tensors defined as follows.

**Definition 1 (Qi, 2005).** Let $\mathcal{A}$ be an *m* th order *n*-dimensional symmetric real tensor. If there exists a nonzero vector *x* ∈ ℝ^*n*^ and a scalar *λ* ∈ ℝ satisfying

(2) \begin{eqnarray*} \left\{ \begin{array}{@{}l@{}} \displaystyle \mathcal{A}{x}^{m-1}=\lambda x, \\ \displaystyle {x}^{T}x=1, \end{array} \right. \end{eqnarray*}

then we call the scalar *λ* a Z-eigenvalue of $\mathcal{A}$, and the vector *x* a Z-eigenvector associated with the Z-eigenvalue *λ*. We also say the pair (*λ*, *x*) is a Z-eigenpair of $\mathcal{A}$.

Iterative algorithms to find the largest or smallest eigenvalues and corresponding eigenvectors are usually designed to solve a nonlinearly constrained optimization problem

\begin{eqnarray*}& \max \nolimits f \left( x \right) =\mathcal{A}{x}^{m} \end{eqnarray*}

(3) \begin{eqnarray*}& s.t.x\in {\mathbb{S}}^{n-1},\end{eqnarray*}

where 𝕊^*n*−1^ denotes the unit sphere in the Euclidean norm, *i.e.,* ${\mathbb{S}}^{n-1}=\{ x\in {\mathbb{R}}^{n}{|}{ \left\| x \right\| }^{2}=1\} $. We can determine the gradient and Hessian of the objective function of Eq. (3) through some simple calculations, as follows:

(4) \begin{eqnarray*}g \left( x \right) \equiv \nabla f(x)=m\mathcal{A}{x}^{m-1}\end{eqnarray*}

and

(5) \begin{eqnarray*}H \left( x \right) \equiv {\nabla }^{2}f \left( x \right) =m \left( m-1 \right) \mathcal{A}{x}^{m-2}\end{eqnarray*}

### Some existing methods for Z-eigenproblems

In this subsection, we introduce some typical methods for computing Z-eigenpairs by solving the problem Eq. (3) or its variants. From Theorem 3.2 of Kolda & Mayo (2011), we know that $ \left( \lambda ,x \right) $ is a Z-eigenpair of $\mathcal{A}$ if and only if *x* is a constrained stationary point of Eq. (3) and $\lambda =\mathcal{A}{x}^{m}/ \left\| x \right\| $. Based on the theorem, De Lathauwer, De Moor & Vandewalle (2000) proposed the S-HOPM method for solving the problem Eq. (3) to find the best symmetric rank-1 approximation of a symmetric tensor $\mathcal{A}\in {\mathbb{S}}^{ \left[ m,n \right] }$, which is equivalent to finding the largest Z-eigenvalue of $\mathcal{A}$ (Qi, 2005). The main step of the S-HOPM algorithm is

(6) \begin{eqnarray*}{x}_{k+1}= \frac{\mathcal{A}{x}_{k}^{m-1}}{ \left\| \mathcal{A}{x}_{k}^{m-1} \right\| } ,{\lambda }_{k+1}=\mathcal{A}{x}_{k+1}^{m}.\end{eqnarray*}

Under the assumption of convexity on $\mathcal{A}{x}^{m}$, S-HOPM could be convergent for even-order tensors. However, it has been pointed out that S-HOPM can not guarantee to converge globally (Kofidis & Regalia, 2002). To address this issue, Kolda & Mayo (2011) modified the objective function to

(7) \begin{eqnarray*}\hat {f} \left( x \right) =\mathcal{A}{x}^{m}+\alpha { \left\| x \right\| }^{m},\end{eqnarray*}

and proposed the SS-HOPM for solving Eq. (3) with the objective function Eq. (7). SS- HOPM has a similar iterative scheme to S-HOPM, but at the same time has a shortcoming in the choice of the shift *α*. To overcome the limitation, the same authors proposed an adaptive method, called GEAP, which is monotonically convergent and much faster than the SS-HOPM method due to its adaptive shift choice of the shift. GEAP is originally designed to calculate generalized eigenvalues (Chang, Pearson & Zhang, 2009) with a positive definite tensor $\mathcal{B}$. The authors also presented a specialization of the method to the Z-eigenvalue problem, which is equivalent to SS-HOPM except for the adaptive shift. The details of the GEAP specialization are briefly summarized in Algorithm 1.

**Table:** 

**Algorithm 1.** GEAP method for the problem Eq. (3) with the objective function Eq. (7)
**Initialization**: Given a tensor $\mathcal{A}\in {S}^{ \left[ m,n \right] }$, an initial vector *x*_0_ ∈ ℝ^*n*^, and a tolerance *ϵ* > 0. Let *β* = 1 if we want to compute the largest Z-eigenvalue, and let *β* = − 1 if we want to compute the smallest Z-eigenvalue. Let *τ* be the tolerance on being positive/negative definite.
**1**: ${x}_{0}\leftarrow {x}_{0}/ \left\| {x}_{0} \right\| $, and ${\lambda }_{0}\leftarrow \mathcal{A}{x}_{0}^{m}$
**For***k* = 0, 1, ⋯ do
**2**: ${H}_{k}\leftarrow m(m-1)\mathcal{A}{x}_{k}^{m-2}$
**3**: ${\alpha }_{k}\leftarrow \beta \max 0,(\tau -{\lambda }_{min} \left( \beta {H}_{k} \right) )/m$
**4:** ${x}_{k+1}\leftarrow \beta (\mathcal{A}{x}_{k}^{m-1}+\alpha {x}_{k})$
**5:***x*_*k*+1_ = *x*_*k*+1_/ ∥ *x*_*k*+1_ ∥
**6**: ${\lambda }_{k+1}\leftarrow \mathcal{A}{x}_{k+1}^{m}$
**7: Break if**|*λ*_*k*+1_ − *λ*_*k*_|<*ϵ*
**End for**
** Output**: Z-eigenvalue *λ* and its associated Z-eigenvector *x*.

GEAP is a simple and effective approach for computing Z-eigenvalues of a symmetric tensor, but it is not guaranteed to determine the largest eigenvalue or the smallest one, which is exactly the goal in some applications. To obtain these extreme eigenvalues with a higher probability, we propose to reformulate the problem Eq. (3) to make its structure similar to a matrix eigenproblem, so that it can be solved by existing methods for matrices that always converge to extreme eigenvalues.

## Proposed Method

### An alternating direction method for Z-eigenproblems

Motivated by that algorithms for solving matrix eigenproblem can always converge to the largest or smallest eigenvalue, we propose to compute extreme eigenvalues by combining the method of solving the matrix eigenproblem and tensor optimization techniques. To this end, we adopt a variable splitting strategy in which we introduce some superfluous variables and equality constraints over these variables. Specifically, the term $\mathcal{A}{x}^{m}$ with even number *m* is rewritten as $\mathcal{A}{x}_{1}^{2}{x}_{2}^{2}\cdots {x}_{p}^{2}$, where *p* = *m*/2, with the equality constraints *x*_*i*_ = *x*_*j*_(*i*, *j* = 1, 2, …, *p*). Therefore, problem Eq. (3) is transformed into the following model:

\begin{eqnarray*}& \max \nolimits \tilde {f} \left( x \right) =\mathcal{A}{x}_{1}^{2}{x}_{2}^{2}\cdots {x}_{p}^{2} \end{eqnarray*}

(8) \begin{eqnarray*}& s.t.{x}_{i}={x}_{j},i,j=1,2,\ldots ,p\end{eqnarray*}

\begin{eqnarray*}& {x}_{i}\in {\mathbb{S}}^{n-1},i=1,2,\ldots ,p. \end{eqnarray*}

When $\mathcal{A}$ is symmetric and conditions *x*_*i*_ = *x*_*j*_ = *x*(*i*, *j* = 1, 2, …, *p*) hold, we can obtain $\mathcal{A}{x}_{1}^{2}{x}_{2}^{2}\cdots {x}_{p}^{2}=\mathcal{A}{x}^{m}$. Using this fact, the equivalence between problems Eqs. (3) and (8) can be easily checked. It is also worthwhile to note that if all variables except *x*_*i*_ are available and those equality constraints are not considered, the problem Eq. (8) reduces to the standard matrix eigenproblem for the matrix $\mathcal{A}{x}_{1}^{2}\cdots {x}_{i-1}^{2}{x}_{i+1}^{2}\cdots {x}_{p}^{2}$.

Directly solving the problem Eq. (8) may be inefficient because its special structure is not considered, and in doing so, it is easy to converge to a locally optimal point, thus the largest or smallest eigenvalue could not be determined. On the other hand, it is comparatively easy to compute extreme eigenvalues for the matrix cases. In Eq. (8), if all variables except *x*_*i*_ are known and those equality constraints are not considered, solving Eq. (8) can exactly get the largest eigenvalue and the corresponding eigenvector of the matrix $\mathcal{A}{x}_{1}^{2}\cdots {x}_{i-1}^{2}{x}_{i+1}^{2}\cdots {x}_{p}^{2}$. Following this observation and the fact that there are many efficient algorithms available for tackling matrix eigenproblems, we propose a simple alternating direction scheme between solving different matrix eigenproblems for the problem Eq. (8). The details of this method are given in Algorithm 2.

**Table:** 

**Algorithm 2** Alternating direction method (ADM) for Eq. (8)
**Initialization**: Given an even-order tensor $\mathcal{A}\in {S}^{ \left[ m,n \right] }$, initial unit vectors *x*_*i*_ ∈ ℝ^*n*^, i =1 , 2, …, *p*, where *p* = *m*/2, and *ϵ* > 0 is the tolerance. Set *x* = *x*_*p*_, $\mathrm{&lambda;}=\mathcal{A}{x}^{m}$, and *δ* as the absolute difference between successive values of *λ*.
**While***δ* > *ϵ*
**For***i* = 1, 2, …, *p* do
**1**: Compute the matrix $A=\mathcal{A}{x}_{1}^{2}\cdots {x}_{i-1}^{2}{x}_{i+1}^{2}\cdots {x}_{p}^{2}$.
**2**: Find the largest or smallest eigenvalue $\tilde {\lambda }$ and the corresponding unit eigenvector *v* of *A* using any eigenvalue algorithm for matrices.
**3**: Update the variable *x*_*i*_ = *v*.
**End for**
** 4: Set***x* = *x*_*p*_ and $\mathrm{&lambda;}=\mathcal{A}{x}^{m}$.
**End while**
** Output**: Z-eigenvalue *λ* and its associated Z-eigenvector *x*.

The main computational cost lies in tensor-vector multiplications $\mathcal{A}{x}_{1}^{2}\cdots {x}_{i-1}^{2}{x}_{i+1}^{2}\cdots {x}_{p}^{2}$ and matrix eigenvalue computations. For an *m*th order *n*-dimensional symmetric tensor $\mathcal{A}$, it costs *O*(*mn*^*m*^) operations to compute the matrix $\mathbf{A}=\mathcal{A}{x}_{1}^{2}\cdots {x}_{i-1}^{2}{x}_{i+1}^{2}\cdots {x}_{p}^{2}$. The cost of computing the largest or smallest eigenvalue of the matrix **A** is (4/3)*n*^3^, which is much less than the products for large *n*. Compared with GEAP, which has similar computations to our method but needs to compute tensor multiplication at least twice in each iteration, our method can save about half of the time because it only needs to calculate tensor multiplication once in each iteration. This can be verified in the numerical experiments in Section 4.

### Specialization of ADM to fourth-order tensors

The proposed ADM transforms the tensor eigenvalue problem Eq. (3) into a series of matrix eigenvalue problems that are easy to solve. For fourth-order tensors, there are two related variables of *x*_1_ and *x*_2_, and the inner iteration can be omitted because *p* = 1. According to the symmetry property of $\mathcal{A}$, we also have $\mathcal{A}{x}_{1}^{2}{x}_{2}^{2}=\mathcal{A}{x}_{2}^{2}{x}_{1}^{2}$. Therefore, it is not necessary to explicitly write out the variable *x*_2_, and the procedure of Algorithm 2 can be simply described, as shown in Algorithm 3, for fourth-order tensors. To better describe the iterative steps, we use *x*_*k*_ to denote a *k*th iterate in Algorithm 3, rather than the splitting variable as in Algorithm 2.

**Table:** 

**Algorithm 3** Specialization of the ADM to fourth-order tensors
**Initialization**: Given a tensor $\mathcal{A}\in {S}^{ \left[ 4,n \right] }$, initial unit vectors *x*_0_ ∈ ℝ^*n*^, and *ϵ* > 0 is the tolerance. Set ${\lambda }_{0}=\mathcal{A}{x}_{0}^{m}$, *k*: = 0, and *δ* as the absolute difference between successive values of *λ*.
**For***k* = 0, 1, 2⋯ do
**1**: Compute the matrix ${A}_{k}=\mathcal{A}{x}_{k}^{2}$.
**2**: Find the largest or smallest eigenvalue $\tilde {\lambda }$ and the corresponding unit eigenvector *v* of *A*_*k*_ using any eigenvalue algorithm for matrices.
**3**: Update the variable *x*_*k*+1_ = *v* and the eigenvalue ${\lambda }_{k+1}=\tilde {\lambda }$.
**4: Break if**|*λ*_*k*+1_ − *λ*_*k*_|<*ϵ*, set *k* = *k* + 1.
**End for**
** Output**: Z-eigenvalue *λ*_*k*+1_ and its associated Z-eigenvector *x*_*k*+1_.

### Convergence analysis

As shown in the main steps of Algorithm 2 and Algorithm 3, the equality constraints in Eq. (8) are not considered in the process of calculation. A natural question arises about whether the algorithms can converge, and furthermore, whether the algorithms can converge to a Z-eigenvalue of $\mathcal{A}$. In this subsection, we handle these issues using properties of extreme eigenvalues and corresponding eigenvectors of matrices. For simplicity, only the convergence property of Algorithm 3 is analyzed. The convergence property of Algorithm 2 can be analyzed in a similar way.

Let *x*_*k*_ denote the *k*th iterate generated by Algorithm 3. According to Steps 2 and 3, in the case of computing the largest Z-eigenvalue of $\mathcal{A}$, *x*_*k*+1_ is the largest eigenvalue of the matrix $\mathcal{A}{x}_{k}^{2}$. Therefore, the quadratic function $q \left( y \right) ={y}^{T} \left( \mathcal{A}{x}_{k}^{2} \right) y=\mathcal{A}{x}_{k}^{2}{y}^{2}$ reaches a maximum value *λ*_*k*+1_ at the point *y* = *x*_*k*+1_ over the unit sphere 𝕊^*n*^, *i.e.,* ${\lambda }_{k+1}=\mathcal{A}{x}_{k}^{2}{x}_{k+1}^{2}\geq \mathcal{A}{x}_{k}^{2}{y}^{2}$ for all *y* ∈ 𝕊^*n*^. At the same time, *x*_*k*_ is the largest eigenvalue of the matrix $\mathcal{A}{x}_{k-1}^{2}$. These results give

(9) \begin{eqnarray*}{\lambda }_{k+1}=\mathcal{A}{x}_{k}^{2}{x}_{k+1}^{2}\geq \mathcal{A}{x}_{k}^{2}{x}_{k-1}^{2}=\mathcal{A}{x}_{k-1}^{2}{x}_{k}^{2}={\lambda }_{k}.\end{eqnarray*}

Here, the second equality holds because of the symmetric property of $\mathcal{A}$. From Eq. (9), we know that the sequence *λ*_*k*_ generated by Algorithm 3 is nondecreasing. On the other side, *λ*_*k*_ is computed by ${\lambda }_{k}=\mathcal{A}{x}_{k-1}^{2}{x}_{k}^{2}$, where *x*_*k*_ ∈ 𝕊^*n*^. Due to the compactness of the unit sphere 𝕊^*n*^, we also know that the sequence *λ*_*k*_ is bounded above. Consequently, *λ*_*k*_ has a unique limit, and we can readily conclude by posing this as a theorem.

**Theorem 1.** Let *λ*_*k*_ be a sequence generated by Algorithm 3. Then the sequence *λ*_*k*_ is nonincreasing and there exists *λ*^∗^ such that *λ*_*k*_ → *λ*^∗^.

While Theorem 1 ensures that Algorithm 3 always terminates in finitely many iterations, theoretically, it cannot ensure that the sequence *λ*_*k*_ converges to a Z-eigenvalue of $\mathcal{A}$ because the equality constraints in Eq. (8) are omitted in the implementation of Algorithm 3. One possible result is the occurrence of cyclic solutions, that is, two consecutive iterates *x*_*k*_ and *x*_*k*+1_ that satisfy $\mathcal{A}{x}_{k}^{2}{x}_{k+1}={\lambda }_{k}{x}_{k+1}$, $\mathcal{A}{x}_{k+1}^{2}{x}_{k}={\lambda }_{k+1}{x}_{k}$, and *λ*_*k*_ = *λ*_*k*+1_. However, this situation is rarely encountered in the numerical experiments presented in the next section. Additionally, how to theoretically avoid this situation is the subject for future research.

## Numerical Experiments

In this section, we present some numerical results of the ADM for computing the largest or smallest Z-eigenvalues of tensors. The proposed ADM is compared with the GEAP method, which is an adaptive shifted power method first proposed by Kolda & Mayo (2014), and the AG method (Yu et al., 2016), which is an adaptive gradient method with inexact stepsize. All experiments are performed in MATLAB R2017a and the Tensor Toolbox (Bader & Kolda, 2012) under a Windows 10 operating system on a laptop with an Intel(R) Core (TM) i7-10510U CPU and 12 GB RAM. In all numerical experiments, we terminate the computation when the absolute difference between successive eigenvalues is less than 10^−10^, *i.e.,* |*λ*_*k*+1_ − *λ*_*k*_| ≤ 10^−10^, or the number of iterations exceeded the maximum number 500.

In our experiments, we use some typical examples from references (Cui, Dai & Nie, 2014; Kofidis & Regalia, 2002; Nie & Wang, 2014) to assess the performance of the proposed method in finding the largest or smallest Z-eigenvalue of a symmetric tensor. All of the largest or smallest Z-eigenvalues in these examples are given in the original literature. Therefore, those desired values are known in advance.

***Example 1*** (Kofidis & Regalia, 2002) Let $\mathcal{A}\in {\mathbb{S}}^{ \left[ 4,3 \right] }$ be the symmetric tensor with entries

\begin{eqnarray*}& & {a}_{1111}=0.2883, {a}_{1112}=-0.0031, {a}_{1113}=0.1973, {a}_{1122}=-0.2485, \end{eqnarray*}

\begin{eqnarray*}& & {a}_{1223}=0.1862, {a}_{1133}=0.3847, {a}_{1222}=0.2972, {a}_{1123}=-0.2939, \end{eqnarray*}

\begin{eqnarray*}& & {a}_{1233}=0.0919, {a}_{1333}=-0.3619, {a}_{2222}=0.1241, {a}_{2223}=-0.3420, \end{eqnarray*}

\begin{eqnarray*}& & {a}_{2233}=0.2127, {a}_{2333}=0.2727, {a}_{3333}=-0.3054.  \end{eqnarray*}

The largest and smallest Z-eigenvalue of the tensor $\mathcal{A}$ are respectively

\begin{eqnarray*}& {\lambda }_{max}=0.8893,{v}_{max}={ \left( 0.6672,0.7160,0.9073 \right) }^{T}; \end{eqnarray*}

\begin{eqnarray*}& {\lambda }_{min}=-1.0954,{v}_{min}={ \left( -0.6447,-0.3357,0.3043 \right) }^{T}. \end{eqnarray*}

**Figure 1:**
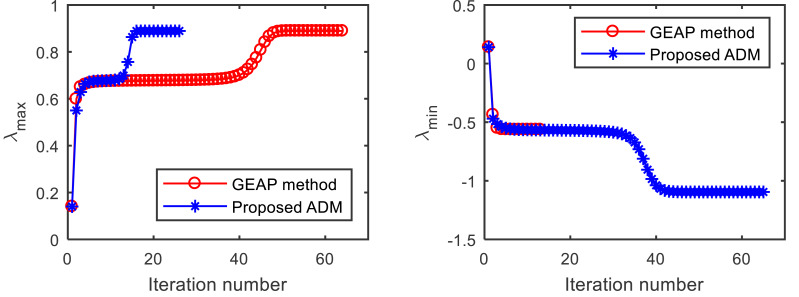
Convergence comparison of the GEAP method and the proposed method for computing the largest and smallest Z-eigenvalue of $\mathcal{A}$ from Example *1*.

We first test the convergence performance of the proposed ADM in comparison to GEAP. Figure 1 shows the convergence trajectories of the two methods for computing extreme Z-eigenvalues of $\mathcal{A}$ from *Example 1*, with the starting point ${x}_{0}={ \left( 0.0417,-0.5618,0.6848 \right) }^{T}$. As shown on the left in Fig. 1, both GEAP and ADM can find the largest Z-eigenvalue 0.8893. Until the stopping criterion is met, GEAP runs 63 iterations in 0.2188 s, while the proposed ADM runs only 26 iterations in 0.0313 s. When computing the smallest Z-eigenvalue with the same starting point (right in Fig. 1), although ADM runs longer than GEAP, the ADM find the desired value of −1.0954, while GEAP fail.

To test the performance of the proposed algorithm in finding extreme eigenvalues, 1,000 random starting guesses drawn uniformly from [-1, 1] are employed in the experiments. All three methods are implemented 1,000 times, each with the same initial point. We list the number of occurrences of extreme eigenvalues (#Occu.), the average number of iterations (Iter_ave_), and the average running time in seconds (CPU_ave_) for the two types of Z-eigenvalues in Tables 1, 2, 3, 4, 5 and 6. As shown in Tables 1–6, for the cases of computing the largest eigenvalues, the proposed ADM runs a similar or slightly larger number of iterations but in a similar or shorter time compared to both GEAP and AG. For the case of computing the smallest Z-eigenvalue, ADM runs slower for *Example 1* but runs much faster for the other examples. It can also be seen from the fourth columns of Tables 1–6 that GEAP can only obtain the largest Z-eigenvalues with a probability of about 0.55, and the smallest Z-eigenvalue with a probability of about 0.65. AG performs clearly better than GEAP. The proposed ADM performs best in almost all cases, which can reach the largest Z-eigenvalues for Examples 2-5 and the smallest Z-eigenvalues for all examples with a probability of 1.

***Example 2*** (Nie & Wang, 2014). Consider the symmetric tensor $\mathcal{A}\in {\mathbb{S}}^{ \left[ 4,n \right] }$ such that

\begin{eqnarray*}{a}_{ijkl}=\sin \nolimits \left( i+j+k+l \right) ,1\leq i,j,k,l\leq n. \end{eqnarray*}

In the case of *n* = 5, the largest and smallest Z-eigenvalues of the tensor $\mathcal{A}$ are respectively

\begin{eqnarray*}& {\lambda }_{max}=7.2595,{v}_{max}={ \left( 0.2686,0.6150,0.3959,-0.1872,-0.5982 \right) }^{T}; \end{eqnarray*}

\begin{eqnarray*}& {\lambda }_{min}=-8.8463,{v}_{min}={ \left( -0.5809,-0.3563,0.1959,0.5680,0.4179 \right) }^{T}. \end{eqnarray*}

***Example 3*** (Cui, Dai & Nie, 2014). Consider the symmetric tensor $\mathcal{A}\in {\mathbb{S}}^{ \left[ 4,n \right] }$ such that

\begin{eqnarray*}{a}_{ijkl}=\tan \nolimits \left( i \right) +\tan \nolimits \left( j \right) +\tan \nolimits \left( k \right) +\tan \nolimits (l),1\leq i,j,k,l\leq n. \end{eqnarray*}

In the case of *n* = 5, we can obtain the largest and smallest Z-eigenvalues of the tensor $\mathcal{A}$ from the reference as follows.

**Table 1:** Comparison results for computing extreme Z-eigenvalues of $\mathcal{A}$ from Example 1.

**Type of Z-eigenvalue**	**Method**	*λ*	**#Occu.**	**Iter**_**ave**_	**CPU**_**ave**_
*λ*_*max*_	GEAP	0.8893	51.00%	27.59	0.0356
	AG	0.8893	**57.60%**	12.97	0.0202
	ADM	0.8893	57.10%	28.96	**0.0165**
*λ*_*min*_	GEAP	−1.0953	41.10%	12.18	**0.0121**
	AG	−1.0953	52.50%	8.24	0.0185
	ADM	−1.0953	**100.00%**	43.52	0.0258

**Note.**

The best results are in bold.

**Table 2:** Comparison results for computing the extreme Z-eigenvalues of $\mathcal{A}$ from Example 2 (*n* = 5).

**Type of Z-eigenvalue**	**Method**	*λ*	**#Occu.**	**Iter**_**ave**_	**CPU**_**ave**_
*λ*_*max*_	GEAP	7.2595	49.80%	49.30	0.0701
	AG	7.2595	60.90%	19.91	**0.0334**
	ADM	7.2595	**100.00%**	117.20	0.0806
*λ*_*min*_	GEAP	−8.8463	51.60%	49.85	0.0692
	AG	−8.8463	77.80%	23.29	**0.0385**
	ADM	−8.8463	**100.00%**	57.41	0.0389

**Note.**

The best results are in bold.

**Table 3:** Comparison results for computing the extreme Z-eigenvalues of $\mathcal{A}$ from Example 3 (*n* = 5).

**Type of Z-eigenvalue**	**Method**	*λ*	**#Occu.**	**Iter**_**ave**_	**CPU**_**ave**_
*λ*_*max*_	GEAP	34.5304	62.30%	27.52	**0.0341**
	AG	34.5304	92.10%	14.54	0.0350
	ADM	34.5304	**100.00%**	56.26	0.0362
*λ*_*min*_	GEAP	−101.1994	72.00%	14.00	0.0184
	AG	−101.1994	98.90%	9.37	0.0149
	ADM	−101.1994	**100.00%**	13.16	**0.0057**

**Note.**

The best results are in bold.

**Table 4:** Comparison results for computing the extreme Z-eigenvalues of $\mathcal{A}$ from Example 4 (*n* = 5).

**Type of Z-eigenvalue**	**Method**	*λ*	**#Occu.**	**Iter**_**ave**_	**CPU**_**ave**_
*λ*_*max*_	GEAP	13.0779	63.20%	22.01	0.0263
	AG	13.0779	93.00%	12.80	0.0209
	ADM	13.0779	**100.00%**	30.72	**0.0166**
*λ*_*min*_	GEAP	−23.5741	69.10%	15.21	0.0161
	AG	−23.5741	97.20%	10.09	0.0193
	ADM	−23.5741	**100.00%**	15.32	**0.0030**

**Note.**

The best results are in bold.

**Table 5:** Comparison results for computing the extreme Z-eigenvalues of $\mathcal{A}$ from *Example 5* (*n* = 5).

**Type of Z-eigenvalue**	**Method**	*λ*	**#Occu.**	**Iter**_**ave**_	**CPU**_**ave**_
*λ*_*max*_	GEAP	9.5821	61.00%	25.59	0.0309
	AG	9.5821	91.70%	14.03	**0.0285**
	ADM	9.5821	**100.00%**	50.69	0.0295
*λ*_*min*_	GEAP	−27.0429	71.60%	13.47	0.0153
	AG	−27.0429	98.40%	9.13	0.0159
	ADM	−27.0429	**100.00%**	12.86	**0.0050**

**Note.**

The best results are in bold.

**Table 6:** Comparison results for computing the extreme Z-eigenvalues of $\mathcal{A}$ from *Example 6*.

**Type of Z-eigenvalue**	**Method**	*λ*	**#Occu.**	**Iter**_**ave**_	**CPU**_**ave**_
*λ*_*max*_	GEAP	1	51.50%	7.56	0.0077
	AG	1	**82.80%**	6.28	0.0054
	ADM	1	64.70%	12.40	**0.0066**
*λ*_*min*_	GEAP	0	99.90%	231.32	0.3365
	AG	0	**100.00%**	22.73	0.0595
	ADM	0	**100.00%**	12.15	**0.0027**

**Note.**

The best results are in bold.

\begin{eqnarray*}& {\lambda }_{max}=34.5304,{v}_{max}={ \left( 0.6665,0.1089,0.4132,0.6070,-0.0692 \right) }^{T}; \end{eqnarray*}

\begin{eqnarray*}& {\lambda }_{min}=-101.1994,{v}_{min}={ \left( 0.2248,0.5541,0.3744,0.2600,0.6953 \right) }^{T}. \end{eqnarray*}

***Example 4*** (Nie & Wang, 2014). Let $\mathcal{A}\in {\mathbb{S}}^{ \left[ 4,n \right] }$ be a symmetric tensor with

\begin{eqnarray*}{a}_{ijkl}=\arctan \nolimits \left( { \left( -1 \right) }^{i} \frac{i}{n} \right) +\arctan \nolimits \left( { \left( -1 \right) }^{j} \frac{j}{n} \right) +\arctan \nolimits \left( { \left( -1 \right) }^{k} \frac{k}{n} \right) +\arctan \nolimits \left( { \left( -1 \right) }^{l} \frac{l}{n} \right) . \end{eqnarray*}

In the case of *n* = 5, the largest and smallest Z-eigenvalues of the tensor $\mathcal{A}$ are respectively

\begin{eqnarray*}& {\lambda }_{max}=13.0779,{v}_{max}={ \left( 0.3174,0.5881,0.1566,0.7260,0.0418 \right) }^{T}; \end{eqnarray*}

\begin{eqnarray*}& {\lambda }_{min}=-23.5740,{v}_{min}={ \left( 0.4403,0.2382,0.5602,0.1354,0.6459 \right) }^{T}. \end{eqnarray*}

***Example 5*** (Nie & Wang, 2014). Let $\mathcal{A}\in {\mathbb{S}}^{ \left[ 4,n \right] }$ be a symmetric tensor with

\begin{eqnarray*}{a}_{ijkl}= \frac{{ \left( -1 \right) }^{i}}{i} + \frac{{ \left( -1 \right) }^{j}}{j} + \frac{{ \left( -1 \right) }^{k}}{k} + \frac{{ \left( -1 \right) }^{l}}{l} ,1\leq i,j,k,l\leq n. \end{eqnarray*}

For *n* = 5, we can get the largest and smallest Z-eigenvalue of the tensor $\mathcal{A}$ with

\begin{eqnarray*}& {\lambda }_{max}=9.5821,{v}_{max}={ \left( -0.1125,0.7048,0.2507,0.5685,0.3233 \right) }^{T}; \end{eqnarray*}

\begin{eqnarray*}& {\lambda }_{min}=-27.0429,{v}_{min}={ \left( -0.6900,-0.1987,-0.4717,-0.2806,-0.4280 \right) }^{T}. \end{eqnarray*}

**Figure 2:**
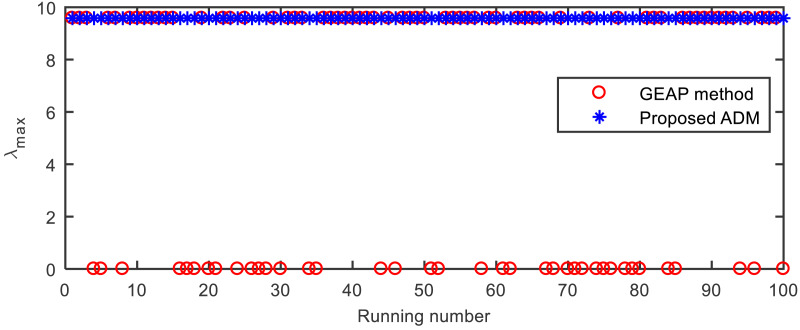
The largest Z-eigenvalues computed by GEAP and ADM in the 100 runs on the tensor $\mathcal{A}$ from Example 5.

Besides the point *λ*_1_ = 9.5821 of the largest Z-eigenvalue, the tensor $\mathcal{A}$ has another stable eigenvalue *λ*_2_ = 0. As shown in Figure 2 and Table 5, GEAP falls into the latter point with a probability of around 0.4, while ADM can always converge to the previous point.

***Example 6*** (Sheng & Ni, 2021). Let $\mathcal{A}\in {\mathbb{S}}^{ \left[ 6,3 \right] }$ be a tensor with

\begin{eqnarray*}{a}_{111122}= \frac{1}{15} ,{a}_{112222}= \frac{1}{15} ,{a}_{112233}=- \frac{1}{30} ,{a}_{333333}=1, \end{eqnarray*}

and *a*_*i*_1_⋯*i*_6__ =0 if (*i*_1_⋯*i*_6_) is not a permutation of an index in the above. We can get the largest Z-eigenvalue *λ*_*max*_ = 1 and smallest Z-eigenvalue *λ*_*min*_ = 0, and these corresponding eigenvectors are not unique. The comparison results are shown in Table 6, from which we find that GEAP converges very slowly when computing the smallest eigenvalue of $\mathcal{A}$. In contrast, ADM reaches the smallest eigenvalue with only about 12 iterations for each execution.

## Discussion

In general, algorithms for computing the largest or smallest eigenvalue of a higher-order tensor are prone to getting stuck in local extrema and then converging to an arbitrary eigenvalue of the tensor depending on the initial conditions. However, the counterpart for symmetric matrices can always converge to the largest or smallest one. Motivated by this, we propose combining algorithms for matrix eigenproblem and tensor optimization techniques in order to obtain extreme eigenvalues. Specifically, the tensor eigenproblem is split into a series of matrix eigenvalue problems using a variable splitting method, and then an alternating scheme is proposed to solve the problem.

To solve the tensor eigenproblems, many algorithms for matrix eigenproblems are extended to the tensor case. However, these generalizations cannot guarantee the low complexity of these algorithms, and the global convergence to the extreme eigenvalues is also not ensured. In this article, the tensor eigenvalue problem is directly transformed into a series of matrix eigenvalue problems so that its algorithms can be directly used to solve the original tensor eigenvalue problem. This method not only overcomes local minima problems existing in direct generalizations, but also has great potential to speed up the convergence. The experimental results verify the effectiveness and advancement of the proposed algorithm, which converges rapidly in most cases and reaches extreme Z-eigenvalues with a significantly higher probability. In many cases, we determine the extreme eigenvalue with a probability of 1, indicating that we can obtain the extreme eigenvalue under any given initial value in these cases. This demonstrates the significant robustness of the proposed method.

However, the proposed algorithm cannot guarantee global convergence for each type of tensor. For Examples 1 and 6, we could only obtain the largest eigenvalue with a probability of about 0.6. In future research, the question of why this kind of tensor cannot obtain the extreme eigenvalue under any initial point will be discussed in more detail.

## Conclusion

In this article, we transform a tensor Z-eigenvalue problem into a series of matrix eigenvalue problems using a variable splitting method and propose an alternating scheme for computing the largest or smallest Z-eigenvalue of symmetric tensors. Just like the classical power method, which constantly uses the intermediate iterates to construct a vector, the proposed algorithm uses them to construct a matrix and computes the eigenvalues and corresponding eigenvectors of the matrix. We analyze the convergence properties of the proposed method which is verified in the numerical experiments. The limitations of this work are twofold. First, as the authors note themselves, it can only ensure the convergence of eigenvalues, but not the convergence of eigenvectors due to the possible existence of cyclic solutions. Second, as can be seen from the numerical examples, it cannot obtain the largest eigenvalues with a 100% probability. The numerical results are reported for some testing examples, which showed that the proposed method converged much faster than both GEAP and AG in most cases and could reach the extreme Z-eigenvalues with a significantly higher probability than GEAP.

##  Supplemental Information

10.7717/peerj-cs.1242/supp-1 File S1 Raw data and Matlab codes

## Additional Information and Declarations

### Data Availability

The following information was supplied regarding data availability: The code is available in Supplementary Files.
